# Leaf and Community Photosynthetic Carbon Assimilation of Alpine Plants Under *in-situ* Warming

**DOI:** 10.3389/fpls.2021.690077

**Published:** 2021-07-08

**Authors:** Zijuan Zhou, Peixi Su, Xiukun Wu, Rui Shi, Xinjing Ding

**Affiliations:** ^1^Key Laboratory of Land Surface Process and Climate Change in Cold and Arid Regions, Northwest Institute of Eco-Environment and Resources, Chinese Academy of Sciences, Lanzhou, China; ^2^Key Laboratory of Desert and Desertification, Northwest Institute of Eco-Environment and Resource, Chinese Academy of Sciences, Lanzhou, China

**Keywords:** alpine meadow, climate warming, photosynthesis, plant communities, up-scaling

## Abstract

The Tibetan Plateau is highly sensitive to elevated temperatures and has experienced significant climate warming in the last decades. While climate warming is known to greatly impact alpine ecosystems, the gas exchange responses at the leaf and community levels to climate warming in alpine meadow ecosystems remain unclear. In this study, the alpine grass, *Elymus nutans*, and forb, *Potentilla anserina*, were grown in open-top chambers (OTCs) for 3 consecutive years to evaluate their response to warming. Gas exchange measurements were used to assess the effects of *in-situ* warming on leaf- and community-level photosynthetic carbon assimilation based on leaf photosynthetic physiological parameters. We introduced a means of up-scaling photosynthetic measurements from the leaf level to the community level based on six easily measurable parameters, including leaf net photosynthetic rate, fresh leaf mass per unit leaf area, fresh weight of all plant leaves in the community, the percentage of healthy leaves, the percentage of received effective light by leaves in the community, and community coverage. The community-level photosynthetic carbon assimilation and productivity all increased with warming, and the net photosynthetic rate at the leaf level was significantly higher than at the community level. Under elevated temperature, the net photosynthetic rate of *E. nutans* decreased, while that of *P. anserina* increased. These results indicated that climate warming may significantly influence plant carbon assimilation, which could alter alpine meadow community composition in the future.

## Introduction

The global average air temperature has increased continuously since the industrial revolution (IPCC, 2013). High-latitude and high-altitude ecosystems are exceptionally sensitive to rising temperatures and experience greater increases in amplitude (Yang et al., 2010). The Tibetan Plateau have experienced rapid climate warming (0.4°C per decade for the past 50 years), exceeding the global mean value, and the warming is expected to increase by 0.6–0.9°C per decade in the 2015–2050 period (Ma et al., 2017). Climate warming has a significant impact on ecosystem carbon cycles, causing both positive and negative feedbacks to future climates (Brient and Bony, 2013). Alpine meadow is a typical vegetation type in the Tibetan Plateau that is fragile and sensitive to human activities and climate change (Peng et al., 2015). A significant component of alpine meadow ecosystems is alpine plants, which are specially adapted to tolerate long-term low temperatures and are highly temperature-responsive (Yu et al., 2010; Che et al., 2014). Climate warming leads to elevated air and soil temperatures, which can directly or indirectly affect plant photosynthesis and growth rates.

Plants are the basis of the carbon cycle. They are not only the source of photosynthetic carbon in ecosystems but are also the key regulators of carbon dioxide (CO_2_) release into the atmosphere from the ecosystem. A large number of simulated warming experiments have demonstrated that climate warming impacts the physiological and ecological characteristics of plants, causing changes in plant phenology, biomass, growth, and reproduction, as well as other characteristics. In cold habitats, temperature is a limiting factor for plant photosynthesis, and species living in these colder regions benefit more from warming than those living in warmer climates (Parmesan, 2007). Due to the temperature dependence of plants, physiological variables such as photosynthesis may be one of the variables most affected by climate change (Luo, 2007). Photosynthesis is the fundamental basis for plant carbon accumulation and is also one of the vital physiological processes easily impacted by environmental changes (Magaña Ugarte et al., 2019). Although leaf temperature may occasionally reach the optimal temperature for photosynthesis in alpine areas, it is typically lower than the optimal temperature throughout most of the growing season, indicating that the photosynthesis of alpine plants is often limited by low temperature (Sáez et al., 2018). Our previous study reported that air temperature is an important factor that affects the photosynthesis of alpine plants (Zhou et al., 2017). Understanding the response of photosynthesis to temperature changes is important for predicting the carbon balance of terrestrial ecosystems and the geographical distribution of vegetation under climate change scenarios.

Photosynthesis is typically described in terms of CO_2_ assimilation capacity, which can be described at the leaf or canopy (or community) level. Community apparent photosynthetic (CAP) reflects the behavior of groups of leaves or individual plants (Gao et al., 2010). Studying CAP can provide a basis for the assessment of the carbon cycle at regional and even global scales. Leaf net photosynthesis provides an overall reflection of physiological processes and is used to compare differences between individuals, while CAP is useful for evaluating competition and mutual benefits among different species. Many studies have discussed the response of leaf and community level photosynthesis to climate change, but warming effects on plant photosynthesis remain controversial. At the leaf level, different species have different responses to warming. Yang et al. (2011) demonstrated that alpine plants displayed a higher photosynthetic capacity and photosynthetic nitrogen use efficiency under warming. Elmendorf et al. (2012) discovered that the response to long-term warming was opposite by grasses, sedges, and rushes. Liang et al. (2013) used a meta-analysis to estimate the effects of warming on leaf photosynthesis of terrestrial plants, and found that the effect of warming on grass was greater than that of forbs, indicating that forbs may accumulate lower biomass and have less competitive than grasses under climate warming. At the community level, some researchers found that warming had no significant effect on gross primary production (GPP) in an alpine meadow on the Qinghai-Tibetan Plateau (Fu et al., 2013), but others thought that experimental warming leading to an increase in GPP in a typical alpine meadow (Peng et al., 2014; Chen et al., 2017). Synchronous observations of carbon assimilation in plants at the leaf and community levels could improve our understanding of the response and adaptability of alpine ecosystems to climate warming.

The gas exchange chamber method is presently commonly used to measure gas exchange at community scales. Models that are used at large spatial scales are typically based on leaf-level gas exchange responses (Lombardozzi et al., 2015; Wehr et al., 2016). CAP can be estimated using canopy-scale models, such as the biochemical modeling of leaf photosynthesis (Farquhar et al., 1980), the big-leaf model (Sellers et al., 1992), the multi-layer model (Leuning et al., 1995), and the two-leaf model (Wang and Leuning, 1998). These models are all based on a model that combines physiological and biochemical indicators (such as phloem migration rate, carboxylation rate, and transport conductance) and involves many parameters and complicated observations (Tang et al., 2015). This led us to speculate on whether leaf photosynthesis and other easily observable parameters could be used to deduce community photosynthesis, and furthermore, the relevant factors that should be considered in the conversion process.

Most studies that have used leaf photosynthesis to evaluate community photosynthesis have focused on crops and forest ecosystems (Ellsworth and Reich, 1993; Seidl et al., 2013; Sanz-Sáez et al., 2017). Ellsworth and Reich (1993) reported that leaf traits such as LMA, N and *A*_max_ per unit area are strongly correlated with the cumulative leaf area above the leaf position in the canopy. Hirose (2005) proposed a functional model that integrates the leaf area, solar radiation, canopy structure, canopy microclimate, and photosynthesis capacity. Zhu et al. (2013) developed a photo-acclimation model that links electron photosynthesis and leaf nitrogen concentration for the distribution of nitrogen in the main photosynthetic proteins in leaves. Song et al. (2017) used canopy architecture, a ray tracing algorithm, and C_3_ photosynthetic metabolism to develop a new integrated canopy photosynthesis model. Singh and Parida (2019) divided canopy into sunlit and shaded with three layers (top–mid–bottom). Based on observations on LAI and light penetration, leaf photosynthesis was then used to compute canopy photosynthesis. In conclusion, all the parameters of these models cannot be obtained by actual measurements or simple arithmetic averages. In the present study, we attempted to find a simplified method for estimating community photosynthesis, and to achieve the up-scaling studies from leaf to community photosynthesis.

The photosynthetic responses of alpine plants to climate change will determine their survival and performance and, consequently, their competitive ability. We hypothesized that the effects of climate warming on the photosynthesis of different alpine plants would differ, which would impact the photosynthesis and species composition of the community. Our objectives were to elucidate the following: (1) the influence of warming on the photosynthesis of an alpine grass and forb; and (2) the relationship between leaf and community photosynthesis using synchronous field observations. The differential responses of different species to photosynthesis under increasing temperature could alter their carbon accumulation, which in turn could impact their competitiveness, community structure, and composition. Therefore, understanding the photosynthetic response at the leaf and community level is critical for predicting the effect of future climate changes and for determining the response mechanism of alpine plants to climate change.

## Materials and Methods

### Study Site

The study site was located at the Zoige Alpine Wetland Ecosystem Research Station (33°51′52″N, 102°08′46′E, 3,440 m) on the eastern Tibetan Plateau. The region has a plateau continental semi-humid climate, with no absolute frost-free period throughout the year. From 1967 to 2010, the annual mean air temperature was 1.7°C and the annual mean precipitation was 600 mm, with 80% of precipitation falling between June and September. The soil is classified as silt clay loam, which is composed of 31.2% sand, 56.0% silt, and 12.8% clay in the top 30 cm soil layer based on the classification of the US Department of Agriculture. Soil organic carbon content, total carbon, and total nitrogen in the top 30 cm layer are 44.5, 46.2, and 4.3 g kg^–1^, respectively. The pH value of the 0–10 cm soil layer is 7.7 (Su et al., 2018). The area is dominated by the perennial plant *Elymus nutans* Griseb., which is an important forage species with high yield and good reproductive capacity. Other common plants include *Potentilla anserina* L., *Roegneria nutans* (Keng) Keng & S. L. Chen, *Poa pratensis* L., *Kobresia setschwanensis* Hand.-Mazz., *Leymus secalinus* (Georgi) Tzvel, *Plantago depressa* Willd., and *Ajania tenuifolia* (Jacq.) Tzvel. These species together account for ∼90% of the aboveground biomass (g m^–2^). In this study, two dominant herbaceous species (*E. nutans* and *P. anserina*) were selected for leaf photosynthesis physiological trait analysis. *Potentilla anserina* is a cosmopolitan species and miscellaneous forb in alpine meadows that is able to reproduce asexually and also exhibits great colonization ability and morphological plasticity.

### Experimental Design

**FIGURE 1:**
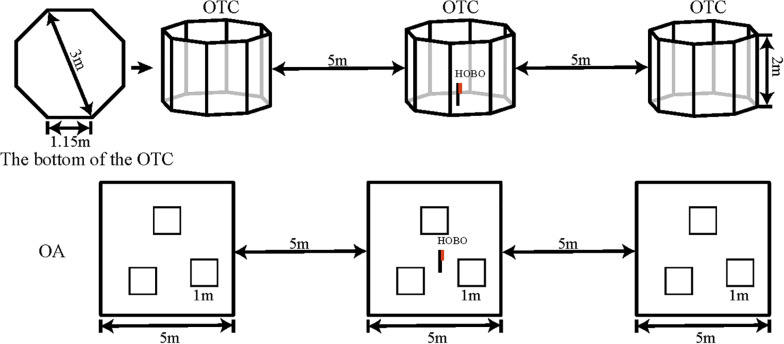
Layout of the experiment design.

In April 2015, three open-top chambers (OTCs) were built to evaluate the effects of warming on the alpine meadow ecosystem. The OTCs possessed underground anti-lateral seepage treatment, and warming can be adjusted by the ancillary facilities. Each OTC comprised an aluminum frame fitted with 8-mm-thick transparent plexiglass boards with a light transmittance >92%. Each unit covered an area of 6.4 m^2^, with a bottom side length of 1.15 m, and was shaped as a regular octagon with an outer diameter of 3 m. The height of OTCs is higher than others (2 vs. 0.5 m) (Li et al., 2019; Chen et al., 2020), and the sides are perpendicular to the ground in order to reduce the effect of precipitation on soil moisture. Window and door were arranged in the north and south sides, which could reduce the amplitude of warming and to avoid excessive overheating in the middle of the day. Open areas (OAs) were established as control areas with similar characteristics to those where the OTC was placed. The layout of the experimental design was shown in Figure 1. We installed microclimate data loggers (HOBO U23-002, RH/Temp Onset, Pocasset, MA, United States) in the center of the OTCs and OAs at 1.5 m height and recorded the air temperature (°C) and relative humidity (%) at 30 min intervals. An ECH_2_O-TE sensor and EM50 data acquisition system were used to monitor the soil temperature and moisture at 5-cm depth at 30 min intervals. Individuals of both species growing in OTCs and OAs were randomly selected for growth measurements and photosynthesis during the growing seasons.

### Leaf Gas Exchange

Due to the late start of the growing season, plants in the study area began to turn green in May and turned yellow and went dormant by late September, and thus the photosynthetic parameters of the plants were usually relatively stable from July to August. The leaf gas exchange parameters of *E. nutans* and *P. anserina* were measured three times per month in July 2016 and 2017. We used a LI-6400 portable photosynthesis system (LI-COR, Lincoln, NE, United States) to measure the leaf net photosynthetic rate (*P*_n_). For *E*. *nutans*, three leaves were fastened side by side to a flat surface with adhesive tape at both ends in order to cover the entire leaf chamber of the infrared gas analyzer and to avoid overlap of the leaves. The leaves were then collected, and their areas were measured with Image J for accurate calculations.

The measurement days were bright and clear with no wind, with hourly measurements from 08:00 to 18:00 h. Measurements were repeated three times. As the local time delay is 72 min from Beijing time, local time was used in the analyses.

### Light and CO_2_ Response of Photosynthesis

To determine the effect of photosynthetic photon flux density (PPFD) levels on photosynthesis, standard light response curves were constructed using a LI-6400 portable photosynthesis system with a LI-6400-02 LED source. An automatic procedure was used to measure *P*_n_ at leaf temperatures of 15, 20, and 25°C, respectively. The ambient CO_2_ concentration (380 μmol mol^–1^) was controlled by the LI-6400 CO_2_ injecting system. The PPFD started at 2,000 and decreased to 0 μmol m^–2^ s^–1^. The response of net photosynthesis CO_2_ uptake (*P*_n_) to varying substomatal CO_2_ concentration (*C*_i_) was determined from *P*_n_–*C*_i_ curves. After pre-induced in leaves for 30 min under saturated light intensity (1500 μmol m^–2^ s^–1^), and followed by the CO_2_ concentration levels from 1,500 to 30 μmol mol^–1^ in turn. The *P*_n_ was measured within 3–5 min to complete the photosynthesis measurement at each CO_2_ level.

Light and CO_2_ responses were taken from 08:00 to 13:30 h during mid-July. Each species was measured in triplicate. Afterward, all the leaves were collected, and the areas were measured with Image J to re-compute the photosynthesis data.

### Community Gas Exchange Measurements

A LI-8100 carbon flux (LI-COR) measurement system and a modified assimilation chamber (0.5 × 0.5 × 0.5 m) (Beijing Ecotek Ltd. Co., Beijing, China) were used to measure community gas exchange. In order to seal the canopy chamber to the soil surface, we installed a 0.5 × 0.5 m square aluminum frame into the soil at a depth of 3 cm, which provided a plane interface between them. During measurements, two small fans were installed diagonally inside the chamber and fanned continuously to mix the atmosphere. The measurements were synchronized with the leaf gas exchange measurements (within three times per month in July 2016 and 2017). The measurements were taken once-hourly between 08:00–18:00 h, using a 5-min measurement that was repeated three times to obtain average values. The CAP rate (μmol CO_2_ m^–2^ s^–1^) was calculated as follows based on the measurement principle of Gao et al. (2010):

(1) $$\text{CAP}=\frac{-{\text{V}}_{\text{A}}\,\times \text{P}\times \,\left(\frac{\partial \text{C}}{\partial \text{t}}-\text{n}\,\frac{\partial \text{Cs}}{\partial \text{t}}\right)}{\text{A }\times \;\,\left(\text{T}+273.15\right)\times R}$$

where A is the total leaf area of the plant canopy (m^2^), V_A_ is the total volume of the community photosynthesis measurement system, which is the product of the height of the assimilation chamber (0.5 m) and the soil surface (0.25 m^3^) equal to 0.125 m^3^, $\frac{\partial \text{C}}{\partial \text{t}}$ is the rate of chamber CO_2_ change (μmol CO_2_ mol^–1^ s^–1^), $\frac{\partial {\text{C}}_{\text{s}}}{\partial \text{t}}$ is the rate of chamber CO_2_ change in soil respiration measurements (μmol CO_2_ mol^–1^ s^–1^), P is the atmospheric pressure (Pa), T is the air temperature inside the chamber (°C), and R is the gas constant (8.314 Pa m^3^ mol^–1^ K^–1^).

### Upscaling the Photosynthesis Measurements From the Leaf to the Community

Alpine meadow plants have obvious stratified structures. The upper layer was dominated by grass, and accounting for nearly 60% of the total coverage. The lower layer was dominated by sedge and forb with over 40% of the total coverage. In this study, we selected *E. nutans* and *P. anserina* to represent the upper and lower layer species. *Elymus nutans* is a grass (Gramineae) and is the dominant species in alpine meadows, while *P. anserina* is a forb (Rosaceae) and is widespread and common. We used the community-weighted mean (CWM) to calculated a given trait (t). CWM_t_ was calculated as follows (Vitra et al., 2019):

(2) $${\mathrm{CWM}}_{t}=\sum_{i=1}^{n}p_{i}\,{\text{x}}_{\text{i}}$$

where CWM_t_ is the community-weighted mean value of a given traits, *p*_i_ is the relative abundance of the ith species (%), *x*_i_ is the mean trait value of species i, and n is the number of species.

Considering the degree of shading between the plants and the angle of the leaves, the parameter *r* (the percentage of received effective light by leaves in the community) was used in the estimation of community photosynthetic capacity. As for the different leaf maturities in the community, we used the parameter *k* to represent the percentage of healthy leaves.

(3) $${\text{CAP}}_{\text{d}}={\text{CWM}}_{p\text{n}}\times {\text{CWM}}_{\text{A}}\times \text{m}\times k\times r\times \text{c}$$

where CAP_d_ is the deduced value of the community photosynthetic rate from leaf level, CWM*p*_n_ is the community-weighted mean value of *P*_n_ (μmol m^–2^ s^–1^), *P*n is the leaf photosynthetic rate with different plants in the community (μmol m^–2^ s^–1^). CWM_A_ is the community-weighted mean value of fresh leaf mass per unit leaf area (m^2^ g^–1^), A was determined as the ratio of the leaf area to its fresh weight. The leaf area of the fresh leaves was analyzed using Image J. m is the fresh weight of all plant leaves in the community (g m^–2^), using weighting method to measurement the fresh leaf weight of all plants in 0.25 m^2^. *k and r* are the correction parameters, and c is the community coverage (%). The three parameters were measured using quadrat survey with a 50 cm × 50 cm frame. All the parameters were measured after community gas exchange measurements with three replicates.

### Plant Biomass

At the end of the growing seasons in 2016–2018 (late September), plant above-ground biomass (AGB) was investigated in the OTCs and OAs. The above-ground plants growing within a 50 × 50 cm quadrat were cut and weighed. Three replicates were tested.

### Statistical Analysis

Analysis of light and CO_2_ response curves involved calculations of the following parameters: *P*_nmax_ (max net photosynthetic rate), *R*_d_ (dark respiratory rate), *AQY* (apparent quantum yield), *LCP* (light compensation point), *LSP* (light saturation point), *V*_cmax_ (maximum carboxylation rate of Rubisco), and *J*_max_ (RuBP regeneration capacity). The light response curve used the modified model of non-rectangular hyperbola (Ye, 2010), and the CO_2_ response curve used Photosynthesis Assistant (Dundee Scientific, Dundee, United Kingdom), which implements a biochemical model describing photosynthetic rate (Farquhar et al., 1980).

All data were analyzed using SPSS 20.0 (Armonk, NY, United States) and the means and standard error (±SE) were computed. We used a paired *t*-test to examine the significance of warming on air temperature, air relative humidity, soil temperature, soil moisture, CAP and the AGB in OTCs and OAs. We used one-way ANOVAs to explicitly assess the effects of the warming on photosynthetic parameters (*P*_n_, *P*_nmax_, *R*_d_, *AQY*, *LCP*, *LSP*, *V*_cmax_, and *J*_max_) and interannual change of AGB. When ANOVA results were significant at *p* = 0.05, differences among the means were tested using Duncan’s multiple range tests. We used linear regression to test the relationships between *P*_n_, CAP, and CAP_d_.

## Results

### Microclimatic Conditions

Air and soil temperatures increased in the OTCs. The mean ground surface temperatures at 1.5 m during the vigorous growth periods were 14.2 and 13.3°C in the OTCs and OAs, and the air temperatures in the OTCs was increased by 0.9°C (*p* < 0.05, Table 1). The mean soil temperatures at 5 cm depth were 17.3 and 16.8°C in the OTCs and OAs, respectively. Air relative humidity and soil water content were all decreased in the OTCs. The mean daily air relative humidity was very similar between OTCs and OAs (78.1 vs. 78.3%, respectively). The soil water content was significantly lower in the OTCs than in the OAs, and were 22.3 and 24.3%, respectively (*p* < 0.05).

**TABLE 1:** Mean daily air temperature (°C) and relative air humidity (%) at 1.5 m aboveground, mean daily soil temperature (°C) and soil moisture (%) at 5 cm depth in OTCs and OAs in growing period.

Treatment	Air temperature (°C)	Air relative humidity (%)	Soil temperature (°C)	Soil volumetric water content (%)
OTCs	14.2 ± 0.3*	78.1 ± 0.9	17.3 ± 0.2	22.3 ± 0.3
OAs	13.3 ± 0.2	78.3 ± 0.8	16.8 ± 0.3	24.3 ± 0.3*

Recorded in open top chambers (OTCs) and open areas (OAs) between 1 July and 31 August. Values are means ± SE. Significant differences between OAs and OTCs according to paired *t*-tests: *p < 0.05.

### Diurnal Variation in Leaf Photosynthesis

**FIGURE 2:**
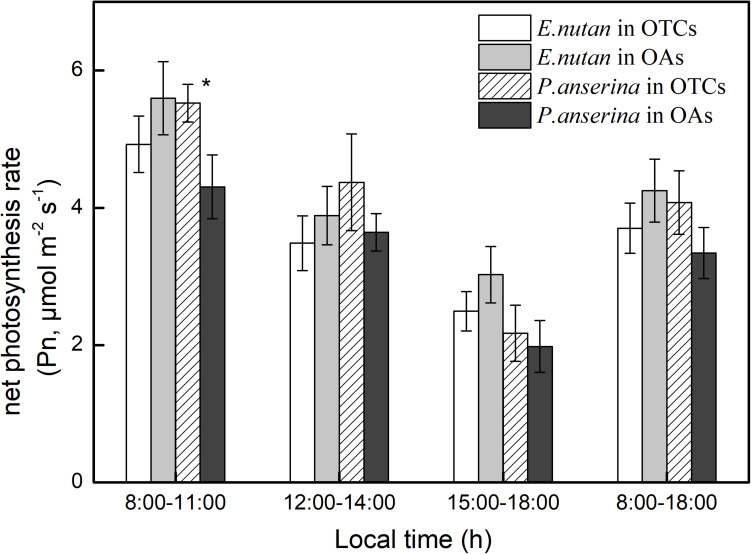
Diurnal changes of net photosynthetic rate (*P*_n_) in alpine meadow at simulated warming. The values are the means ± SE, and are the average of 2 years. * represents a significant correlation at the 0.05 level.

Each measurement day was divided into three periods, including the morning (8:00–11:00), noon (12:00–14:00), and the afternoon (15:00–18:00). In July, the diurnal changes in leaf photosynthesis of the two dominant species were similar, gradually decreasing from 8:00 to 18:00 (Figure 2). In the OAs, the *P*_n_ of *E. nutans* was higher than that of *P. anserina*. Warming decreased the *P*_n_ of *E. nutans* but increased that of *P. anserina*. For *E. nutans*, the daily *P*_n_ average in the OTCs and OAs was 3.7 and 4.3 μmol m^–2^ s^–1^, and the daily *P*_n_ average of *P. anserina* in the OTCs and OAs as 4.1 and 3.3 μmol m^–2^ s^–1^, respectively. The maximum *P*_n_ for *E. nutans* and *P. anserina* occurred at 9:00 AM local time.

### Light and CO_2_ Response Characteristics Under Different Temperatures

**FIGURE 3:**
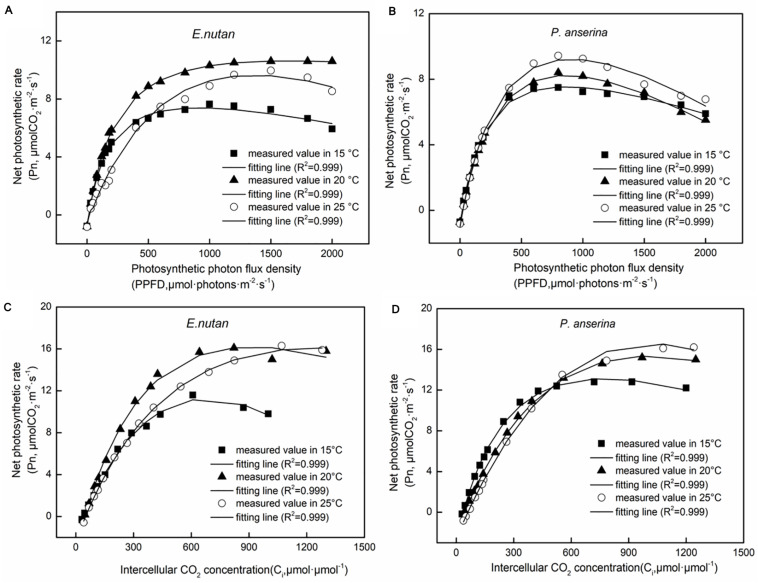
Net photosynthetic rate (*P*_n_) responses to different photosynthetic photon flux density (PPFD) in *E. nutan* **(A)** and *P. anserina* **(B)**, and to different intercellular CO_2_ concentration (C_i_) in *E. nutan* **(C)** and *P. anserina* **(D)**. (mean ± SE; *n* = 3).

Significant differences in photosynthesis parameters between the OAs and OTCs were detected in both species. A higher *P*_nmax_ and *LSP* were recorded in *E. nutans* (Figure 3 and Table 2). At 15°C air temperature, the *P*_nmax_ of *E. nutans* and *P. anserina* were 7.39 and 7.34 μmol m^–2^ s^–1^, while at 20°C air temperature, *E. nutans* had the highest *P*_nmax_, LSP, *R*_d_, and *AQY* values. With the increase in air temperature to 25°C, the *P*_nmax_, *LSP*, *R*_d_, and *AQY* decreased, while the *LCP* increased in comparison to 20°C in *E. nutans*. In *P. anserina*, the increased air temperature was associated with increased *P*_nmax_, *LCP*, *LSP*, and *R*_d_.

**TABLE 2:** Light and CO_2_ response characteristics under different temperatures.

Plants	*T*_a_	*P*_nmax_/(μmol m^–2^ s^–1^)	*LCP*/(μmol m^–2^ s^–1^)	*LSP*/(μmol m^–2^ s^–1^)	*R*_d_/(μmol m^–2^ s^–1^)	*AQY*/(mol mol^–1^)	*V*_cmax_/(μmol m^–2^ s^–1^)	*J*_max_/(μmol m^–2^ s^–1^)
*E. nutans*	15°C	7.39 ± 0.52d	12.73 ± 2.18e	909.3 ± 56.7c	0.68 ± 0.03c	0.057 ± 0.01a	17.14 ± 0.83d	38.33 ± 1.07d
	20°C	10.63 ± 1.25a	15.05 ± 1.28d	1632.1 ± 83.9a	0.86 ± 0.04ab	0.061 ± 0.01a	30.84 ± 0.97b	51.36 ± 1.84b
	25°C	9.65 ± 0.49b	26.44 ± 3.70a	1365.1 ± 33.5b	0.61 ± 0.03c	0.042 ± 0.02c	33.63 ± 1.15a	53.84 ± 1.27a
*P. anserina*	15°C	7.34 ± 0.56d	17.37 ± 1.37c	863.7 ± 11.9c	0.84 ± 0.04ab	0.051 ± 0.01b	23.14 ± 2.18c	50.01 ± 3.29b
	20°C	8.24 ± 0.82c	18.68 ± 1.95c	863.3 ± 10.6c	0.73 ± 0.03bc	0.041 ± 0.01c	24.83 ± 2.59c	45.82 ± 4.79c
	25°C	9.23 ± 0.34b	24.00 ± 0.95b	905.4 ± 23.0c	1.00 ± 0.06a	0.044 ± 0.01c	34.20 ± 2.32a	49.96 ± 2.03b

*Values are means ± SE (*n* = 3). Significant differences in different temperature for each species were according to Duncan test, values with the same letters within columns are not significantly different at *p* < 0.05. *T*_*a*_, air temperature; *P*_*nmax*_, max net photosynthetic rate; *R*_*d*_, dark respiratory rate; *AQY*, Apparent quantum yield; *LCP*, Light compensation point; *LSP*, Light saturation point. *V*_*cmax*_, maximum carboxylation rate of Rubisco; *J*_*max*_, RuBP regeneration capacity.*

From 15 to 25°C, the *V*_cmax_ and *J*_max_ of *E. nutans* increased significantly (Figures 3C,D and Table 3, *p* < 0.05], while in *P. anserina, J*_max_ was highest at 15°C.

**TABLE 3:** Upscaling of the photosynthesis from leaf to community.

Treatments	CWM*p*_n_ (μmol m^–2^ s^–1^)	CWM_A_ (m^2^ g^–1^)	m (g m^–2^)	*k*	*r*	c (%)	CAP (μmol m^–2^ s^–1^)	CAP_d_ (μmol m^–2^ s^–1^)
OTCs	3.87 ± 0.33	(6.80 ± 0.25) × 10^–3^	164.8 ± 5.2	0.80 ± 0.04	0.63 ± 0.06	96 ± 3	2.05 ± 0.18	2.04 ± 0.25
OAs	3.84 ± 0.32	(6.47 ± 0.31) × 10^–3^	151.1 ± 4.8	0.75 ± 0.06	0.65 ± 0.08	94 ± 4	1.59 ± 0.17	1.66 ± 0.20
Average	3.86 ± 0.33	(6.63 ± 0.17) × 10^–3^	158.0 ± 4.3	0.77 ± 0.05	0.64 ± 0.06	95 ± 4	1.82 ± 0.18	1.85 ± 0.16

*Values are means ± SE (*n* = 22). CWM*p*_*n*_, the community-weighted mean value of *P*n; CWM_*A*_, the community-weighted mean value of fresh leaf mass per unit leaf area; *m*, fresh weight of all plant leaves in the community; *k*, percentage of healthy leaves; *r*, percentage of received effective light by leaves in the community; *c*, community coverage; CAP, canopy photosynthesis; CAP_*d*_, deduced value of the community photosynthetic.*

### Diurnal Variation in Alpine Meadow Community Photosynthesis

**FIGURE 4:**
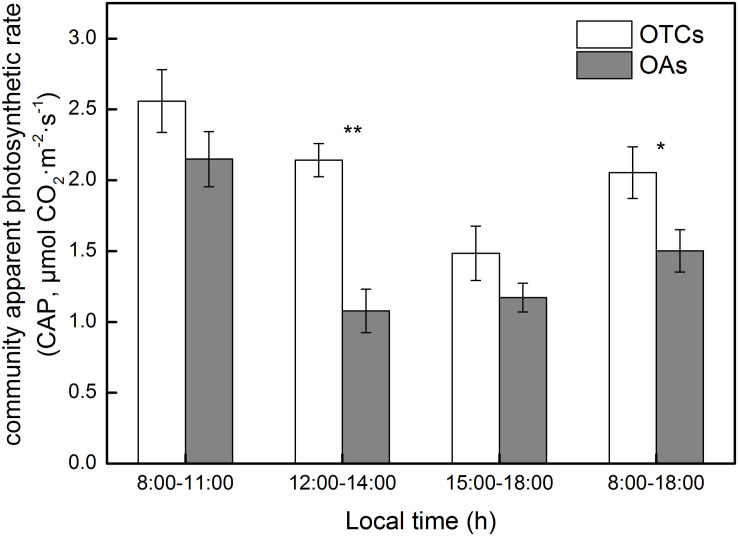
Diurnal changes of community apparent photosynthetic rate (CAP) in alpine meadow at simulated warming. The values are the means ± SE, and are the average of 2 years. * represents a significant correlation at the 0.05 level; ** represents a very significant correlation at 0.01 level.

The diurnal variation in the photosynthetic rate in the alpine meadow community exhibited a decreasing trend. Inside the OTCs, the average CAP was greater than in the OAs (Figure 4, 2.1 μmol CO_2_ m^–2^ s^–1^ in OTCs and 1.6 μmol CO_2_ m^–2^ s^–1^ in OAs). The maximum values of CAP in the OTCs and OAs were observed at 10:00 AM with 3.3 and 2.9 μmol CO_2_ m^–2^ s^–1^, respectively. The simulated warming increased the net photosynthetic rate of the alpine meadow communities by up to 48%, and the difference is significantly (*p* < 0.05).

### Upscaling and Correlation of the Photosynthesis From the Leaf to Community Level

**FIGURE 5:**
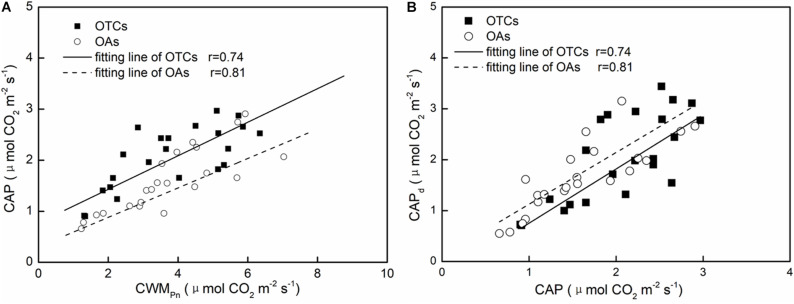
The linear fitting of leaf photosynthetic rate (*P*_n_) and community apparent photosynthetic rate (CAP) **(A)**, and the derived CAP and the measured CAP **(B)** under *in-situ* warming.

We used CWM*_*P*_*_n_ to represent the leaf level photosynthesis with different plants in the community. Linear fitting of the net photosynthetic rate of the leaves and communities of the alpine plants indicated a significant positive correlation between the leaves and communities under *in-situ* warming. The net photosynthetic rate of the community was significantly lower than that of the leaves. The CAP in the OTCs and OAs accounted for 53.0 and 41.4% of the leaf photosynthetic rate, respectively. The overall regression equation for the net photosynthetic rate of the leaves and communities was: CAP = 0.311 CWM*_*P*_*_n_ + 0.659 (*n* = 22, *R* = 0.74, *p* < 0.05) (Figure 5A). It can be seen from Figure 5B and Table 3 that there was a significant correlation between the derived CAP and the measured CAP. The derived value in the OTCs was lower than the measured value, while the derived value in the OAs was greater than the measured value. The difference in CAP between the derived value and the measured value was generally not significant (0.03 μmol CO_2_ m^–2^ s^–1^).

### Biomass Changes in Alpine Meadow Ecosystems

**FIGURE 6:**
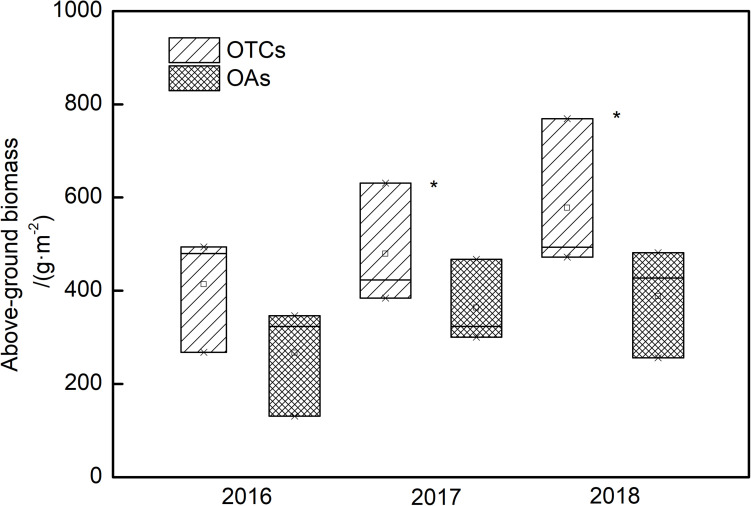
Above-ground biomass (AGB) changes in alpine grassland under simulated warming in 2016–2018. * represents a significant correlation at the 0.05 level among years.

Changes in AGB were observed under simulated warming (Figure 6). After three consecutive years of elevated temperature, the AGB of the alpine vegetation increased. In 2016, the AGB with an average of 413.7 and 266.8 g m^–2^ in the OTCs and OAs, respectively. The differences of AGB were not significant between OTCs and OAs, but a significant increase in biomass was observed in different years (*p* < 0.05). In 2018, a more significant increase in biomass was observed in the OTCs with an average of 578.0 g m^–2^.

## Discussion

### *In-situ* Warming and Its Differential Effect on the Photosynthetic Performance of Two Dominant Species

Climate change is altering the structure and function of alpine ecosystems. The air temperature of the Tibetan Plateau is forecast to increase by 0.6–0.9°C from 2015 to 2050 (Ma et al., 2017), which is within the simulated warming in our experimental setup (Table 1). Under certain circumstances, warming can meet the growth requirements of plants. However, it can also change the microclimatic environment of the plant community and directly or indirectly affect plant photosynthetic physiological processes in a variety of ways. Warming increase the temperature of air and soil, and reduce soil water content, finally leading to dramatic ecological effects on plants (Allison et al., 2018; Samaniego et al., 2018). Many studies found that warming-induced decrease in soil moisture might be the major reason of photosynthesis inhabitation (Reich et al., 2018; Shen et al., 2020). In our study, the decrease of soil moisture in OTCs did not cause soil drought (22.3%), so the presence of an OTC was not an additional source of drought stress (Pugnaire et al., 2020).

Photosynthesis is a key process for the development and carbon assimilation of plants, and can directly influence the productivity and fitness of plants (Lin et al., 2015). Although increased temperature in cold ecosystems, such as our study area, may promote plant growth (Figure 6), it may also increase interspecific competition. The responses of different plants to climate warming differ, and these responses determine the adaptive capacity of species to future climate warming as well as their competitive ability (Elmendorf et al., 2012). In our study, *P*_n_ in *E. nutans* was higher than *P. anserina* in the OAs (Figure 2). Interestingly, the *P*_n_ of *E. nutans* decreased with increased temperature, while that of *P. anserina* increased. Shen et al. (2020) also found that the *P*_n_ of *L. secalimus* significantly decreased under warming, suggesting that warming can negatively limit plant photosynthesis. This is because the air temperature was high in the growing season (July), and thus the leaf temperature of *E. nutans* exceeded its optimal temperature, leading to a decrease in *P*_n_. However, *E. nutans* could provide shading for *P. anserina* due to their taller individuals, and thus resulted in lower heat stress for *P. anserina* growth.

Photosynthetic parameters are very important for estimating the alpine C budget. Previous studies showed that warming would increase plant C uptake by providing optimal temperature conditions (Sage and Kubien, 2007; Niu et al., 2008). In our study, *P. anserina* had a higher photosynthetic rate than *E. nutans* under warming (Figures 2, 3). Shi et al. (2015) suggested that forbs (*Vicia unijuga* and *Allium atrosanguineum*) would adapt better to future climate warming than grasses (*E. nutans* and *Koeleria macrantha*) in alpine meadows, which is consistent with our findings. In cold climates or in areas with no water restrictions, species will change their optimal photosynthetic temperature to increase photosynthesis under warming (Gunderson et al., 2010; Peñuelas et al., 2013). A temperature increase from 15 to 20°C resulted in increased *P*_nmax_, *LSP*, *R*_d_, and *AQY* in *E. nutans*, while a decrease was observed at 25°C. This suggests that 20°C is the approximate optimum growth temperature for *E. nutans*. Conversely, *P*_nmax_, *LCP*, *LSP, R*_d_, and *V*_cmax_ all increased with increased temperature in *P. anserina*, which implies that *P. anserina* can survive at a higher temperature. Similar responses have also been reported by Shi et al. (2010), who found that elevated temperature increased the photoinhibition of *E. nutans* but reduced the photoinhibition of *P. anserina*. In the present study, *E. nutans* demonstrated the highest *P*_nmax_ at 20°C, which thereafter decreased at 25°C, but was still higher than in *P. anserina*. A higher *P*_nmax_ is associated with higher photosynthetic gain, suggesting that *E. nutans* had a greater photosynthetic gain. *E. nutans* have greater photosynthetic gain, while *P. anserina* can survive at a higher temperature, suggesting that the community structure of alpine meadow may change from grass to forb with climate warming. CO_2_ utilization during photosynthesis is indicative of photosynthetic efficiency, and a higher *R*_d_ is indicative of greater consumption of photosynthetic products. *V*_cmax_ and *J*_max_ increased with increased temperature in both species, which might be related to the changes in nitrogen distribution and photosynthetic enzyme activity in the leaves (Fauset et al., 2019).

### Community Photosynthetic Carbon Assimilation and Its Relationship With Leaf-Level Carbon Assimilation

The leaf is the smallest unit of a plant community, and a plant community is the basic component of an ecosystem. CAP represents the photosynthesis of both the top and bottom leaf layers. In the present study, we offer a simplified approach for estimating community photosynthesis. We used six easily measurable parameters to calculate the community photosynthesis and compared these with the observed results (Eq. 3, Figure 5). Ellsworth and Reich (1993) suggested that LMA is an effective means of combining the effects of canopy structure and light environment on leaf photosynthetic performance. In our study, we used fresh leaf mass per unit leaf area (A) and fresh weight of all plant leaves (m) in the scale conversion. Using our equation, the result agrees with the observations under natural conditions (Table 3).

Leaf position, leaf age, and different leaf orientations or growth angles (horizontal and vertical) also influence the photosynthetic rate of the leaves (Zhang et al., 2005). Alpine meadow plants have obvious stratified structures. Grasses (such as *E. nutans*) largely grow in full-sun conditions, while sedges and forbs (such as *P. anserina*) mostly grow in shaded environments. In the canopy, the light absorbed by the upper leaf layer is usually more than its saturation level, and the excess light energy is dissipated primarily by heat dissipation, while the lower layer leaves are usually limited by available light (Keenan and Niinemets, 2016). Considering the degree of shading between the plants and the angle of the leaves, the parameter *r* (the percentage of received effective light by leaves in the community) was used in the estimation of community photosynthetic capacity. As for the different maturity of leaf maturities in the community, we used the parameter *k* to represent the percentage of healthy leaves, and these parameters were successfully incorporated in the model.

In alpine regions, lower temperatures and a short growing season are the main limiting factors for plant growth and ecosystem productivity. Studies have shown that in temperature-limited ecosystems, the extension of the growing season under long-term warming will significantly improve net primary productivity by increasing photosynthetic capacity (Jarvis et al., 2004; Xu et al., 2012). Peng et al. (2017) concluded that alpine ecosystems with low temperature and relatively high soil moisture tend to absorb more C in a warmer climate. AGB is a comprehensive index to reflect growth status and adaptive capacity of plants (Xu et al., 2015; Sanaei et al., 2018). In our study, the AGB of the alpine vegetation increased under three consecutive years of warming, which is consistent with the increase in community photosynthesis under warming conditions (Figure 4). Climate warming increased the AGB of the arctic willow *Salix arctica*, which had a positive feedback on its photosynthetic activity (Sullivan et al., 2008). Under warming, the tested alpine meadow plants increased their AGB due to the increased community photosynthetic rate, which is consistent with Chapin et al. (1995).

## Conclusion

This study linked leaf- and community-level photosynthetic parameters and revealed the effects of warming on the photosynthesis and productivity of alpine plants. The responses of different plants to climate warming differ, which is associated with differential adaptability and competitiveness. Under a temperature increase from 15 to 25°C, the net photosynthetic rate of *E. nutans* increased at 20°C and then decreased, suggesting that 20°C is the optimum growth temperature for *E. nutans*. For *P. anserina*, an increase in air temperature was associated with increased photosynthetic capacity, suggesting that *P. anserina* may have a wider range of temperature adaptations. The net photosynthetic rate of the community was significantly lower than that of the leaves. Under climate warming, the photosynthetic capability and productivity of the alpine meadow communities all increased, suggesting that an increase in temperature under climate warming may have a significant influence on net plant C uptake. Furthermore, we used six easily measurable parameters to scale up from leaf-level to community-level photosynthesis, and the difference in CAP between the derived value and the measured value was not significant.

## Data Availability Statement

The datasets generated for this study are available on request to the corresponding author.

## Author Contributions

ZZ and PS designed the experiments and measured the gas exchange. XW made the data analysis. RS and XD collected the plant samples and measured the leaf traits. All the authors read and approved the final manuscript.

## Conflict of Interest

The authors declare that the research was conducted in the absence of any commercial or financial relationships that could be construed as a potential conflict of interest.
